# Understanding kinesiophobia in pediatric bone tumors: investigating its presence and predictive factors

**DOI:** 10.1007/s00431-025-06032-9

**Published:** 2025-02-13

**Authors:** Güleser Güney Yılmaz, Müberra Tanrıverdi, Gözde Önal, Ayşenur Baysal Yiğit, Sedef Şahin, Fatma Betül Çakır

**Affiliations:** 1 Department of Occupational Therapy, Faculty of Health Sciences, Hacettepe University, Ankara, Türkiye; 2 Department of Physiotherapy and Rehabilitation, Faculty of Health Sciences, Bezmialem Vakıf University, Istanbul, Türkiye; 3 Department of Occupational Therapy, Faculty of Health Sciences, Medipol University, Ankara, Türkiye; 4 Department of Occupational Therapy, Faculty of Bor Health Sciences, Niğde Ömer Halisdemir University, Niğde, Türkiye; 5 Department of Pediatric Hematology and Oncology, Faculty of Medicine, Bezmialem Vakıf University, Istanbul, Türkiye

**Keywords:** Tampa Scale for Kinesiophobia (TSK), Children Depression Rating Scale-Revised (CDRS-R), Numerical rating scale (NRS), Chronic postsurgical pain (CPSP)

## Abstract

Primary malignant bone tumors are significant health concerns in children. These tumors, often accompanied by pain, fatigue, and reduced physical function, can lead to the development of kinesiophobia, a fear of movement that can further complicate rehabilitation. Although factors associated with kinesiophobia have been examined in various adult cancer populations, there is limited research on kinesiophobia and its predictors in children with bone tumors. This study aims to investigate the factors contributing to kinesiophobia in pediatric bone tumor patients. This prospective cross-sectional study was conducted in children with primary malignant bone tumors aged 8–17 years who actively on treatment. The Tampa Scale for Kinesiophobia (TSK) was used to assess fear of movement. Other assessments included the numerical rating scale (NRS) for pain, the PedsQL™ Multidimensional Fatigue Scale for fatigue, the Children Depression Rating Scale-Revised (CDRS-R) for depression, manual muscle testing, and the TUG Test for functionality. Logistic regression was performed to identify predictors of kinesiophobia, while chi-square tests examined the relationship between muscle strength and kinesiophobia levels. One hundred children with bone tumors an average age of 11.83 years participated in the study. The logistic regression model indicated that surgery status, pain levels, and fatigue were significant predictors of kinesiophobia, with an *R*^2^ value of 0.870, explaining 87% of the variance in kinesiophobia levels. Children who had surgery and were in more pain and had higher levels of depression were more likely to exhibit kinesiophobia. *Conclusion*: This study highlights the multifactorial nature of kinesiophobia in children with malignant bone tumors, emphasizing the roles of surgical status, pain, and psychological factors. Integrating biopsychosocial assessments and approaches into routine care may be important to reduce kinesiophobia, improve rehabilitation outcomes, and increase overall well-being.

**Table:** 

**What is Known:**	
*• Kinesiophobia has been studied in adult cancer populations and has been associated with decreased physical activity and poorer rehabilitation outcomes.*	
**What is New:**	
*• This study demonstrates that surgery status, pain levels, and fatigue are significant predictors of kinesiophobia in children with malignant bone tumors and highlights that, in addition to various cancer-related symptoms, kinesiophobia can also be present in this population.*

## Introduction

Primary malignant bone tumors, characterized by clinical and biological heterogeneity, form a group of neoplasms originating in the bone and associated tissues [1, 2]. These tumors, particularly osteosarcoma and Ewing sarcoma, are more commonly observed in adolescents and account for 3–5% of childhood cancers [3]. Due to their nonspecific symptoms, poor prognosis, and high morbidity rates, malignant bone tumors represent a significant public health issue globally, especially in developing countries with limited medical resources [4].

Despite being rarer than adult cases, at least 300,000 new childhood cancer cases are reported worldwide each year [5]. As survival rates improve, enhancing children’s quality of life during and after treatment has become a key goal. Treatments like chemotherapy, radiotherapy, and surgery can cause muscle weakness, reduced bone density, and diminished physical function. They may also lead to complications such as immune reactions, infections, fatigue, nausea, and vomiting, commonly seen during therapy [6]. These complications, along with highly variable clinical conditions, can negatively affect patients’ quality of life and lower their treatment tolerance [7]. Prolonged isolation and extended hospital stays also lead to physical inactivity, increasing the risk of additional complications [6].

Children with bone tumors often experience chronic or acute pain due to the disease itself, malignant tumor lesions, and various treatment options [8]. Cancer-related pain has been reported in approximately 67–80% of children due to treatment interventions and in 20–33% directly due to malignancy, leading to severe perioperative acute pain during the surgical period in children undergoing cancer-related surgeries [9, 10]. Pain, the agents used during treatment, surgical interventions, fatigue, and prolonged hospital stays in isolation can lead to movement avoidance in adolescent cancer patients. This avoidance behavior may, in turn, result in the development of kinesiophobia [8, 11]. Kinesiophobia, defined as the “fear of movement,” is typically a fear of re-injury that develops after a painful injury, leading to a reduction in physical activity and movement [12]. Patients with kinesiophobia develop the belief that movements will lead to re-injury and pain. Over time, this leads to decreased physical activity, avoidance of daily tasks, functional decline, reluctance to use extremities, and in advanced stages, may result in depression and reduced quality of life [13].

Research often shows that kinesiophobia mainly stems from cognitive changes like pain-related fear and catastrophizing [14]. A study conducted in Spain reported that preoperative pain catastrophizing and pain anxiety were associated with the duration of pain in children experiencing long-term postoperative pain [15]. The cognitive fear-avoidance model suggests that pain-related fear triggers escape behaviors, leading to activity avoidance. This cycle prolongs, resulting in reduced movement, disability, and depression [16]. Although kinesiophobia is known to be present in various disease groups such as chronic low back pain, fibromyalgia, osteoarthritis, migraine, coronary artery disease, systemic lupus, and Parkinson’s disease, there is very little research available on cancer and kinesiophobia [17–19]. Previous studies have shown that approximately 40% of pain-related disability can be associated with kinesiophobia [20]. For example, it has been suggested that kinesiophobia in patients with breast cancer increases the risk of lymphedema, depression, and poorer upper extremity function [21]. In a study focusing on pediatric populations, chronic postsurgical pain (CPSP) was found to persist in approximately 35–38% of children 6–12 months after surgery and was associated with functional disability and psychological issues [22]. Research examining the relationship between kinesiophobia and general health status in 1236 cancer survivors found that fear of movement is significantly related to general health status. The article also reports that kinesiophobia significantly decreased in individuals with high kinesiophobia after gradual activity and rehabilitation [23].

The relationship between kinesiophobia and bone tumors may be crucial to study, especially given the challenges faced by these patients. Kinesiophobia frequently occurs in those with chronic pain, leading them to avoid movement due to fear of pain or re-injury, which reduces physical activity. This can increase morbidity and decrease quality of life [18]. Bone tumors often cause significant pain, leading patients to avoid movement to prevent discomfort. This fear can be particularly detrimental, as immobility hinders postoperative rehabilitation, limits functional recovery, and worsens physical condition. Kinesiophobia can delay recovery, and if it results in a sedentary lifestyle, it may further decline overall health. A systematic review of kinesiophobia in chronic musculoskeletal pain indicates a strong association with increased pain, disability, and reduced quality of life [8]. These findings suggest that similar mechanisms may affect bone tumor patients, potentially delaying recovery and leading to poorer results.

Understanding the link between bone tumors and kinesiophobia, along with its influencing factors, is crucial to addressing the physical and psychological challenges faced during treatment. In chronic musculoskeletal pain, kinesiophobia is closely tied to increased pain, disability, and reduced quality of life. Similar mechanisms may affect bone tumor patients, potentially hindering treatment outcomes. This study aims to examine the presence of kinesiophobia and predictors of kinesiophobia in children with malign pediatric bone tumors.

## Method

Our prospective cross-sectional study included patients hospitalized at the university’s medical faculty training and research hospital who had a stable medical condition suitable for the evaluation parameters. The patients had been diagnosed by a pediatric hematologist-oncologist. They were hospitalized for active treatment. The outcomes used were applied to patients selected by the pediatric hematologist-oncologist, ensuring no risk of discomfort during the evaluation process. The sample size estimation was performed using G*Power 3.1.9.4. The Tampa Scale of Kinesiophobia score being a purely kinesiophobia assessment, was chosen for power analysis to determine required minimal sizes of our study. Based on a study, the minimal sample size was estimated to be *N* ≥ 100 participants using G*Power software to obtain a power of 0.80 (*β* = 0.20) with *α* = 0.05 (*p* < 0.05) with an effect size of 0.85 [24]. The inclusion criteria were as follows: (1) being between 8 and 17 years old, (2) having a diagnosis of a malignant bone tumor, (3) being in a condition that does not prevent them from comprehending and completing the questionnaires independently, and (4) surviving for more than 6 months. The exclusion criteria were (1) being in a palliative care process, (2) having a serious neurological or psychiatric diagnosis, including severe neurological or psychiatric disorders, (3) having any other type of tumor or metastasis, and (4) having severe infection or instability in children undergoing surgery.

### Procedure

The study was conducted with children after surgery admitted to the university’s hematology and oncology service at March–July 2024. The data collected prospectively; the study was cross-sectional. The TAMPA Kinesiophobia Scale was used to assess the level of kinesiophobia.

### Assessments

#### Demographic form

A demographic form was administered to collect detailed information about the children’s diagnosis, age, BMI, gender, surgical history, treatment processes, and other related details.

#### Tampa scale of kinesiophobia

The TSK [14] is a 17-item scale designed to measure fear of movement-evoked pain and injury. Total scores range from 17 to 68, with higher scores indicating greater fear of movement. The TSK has demonstrated good internal consistency, with Cronbach’s alpha values ranging from 0.68 to 0.86 [25, 26]. The validity and reliability of the test have also been established in children, making it suitable for use in this population as well [27]. The Turkish Tampa Scale of Kinesiophobia was applied in the study [28].

**PedsQL™ Multidimensional Fatigue Scale (PedsQL™ MFS)** is a widely used tool for assessing fatigue in pediatric patients. Developed by Varni [29], this instrument is based on the concept that disease-specific symptoms are key indicators of general health-related quality of life. The 18-item scale is divided into three subscales: general fatigue, sleep/rest fatigue, and cognitive fatigue. The PedsQL Multidimensional Fatigue Scale (PedsQL™ MFS) showed satisfactory to good internal consistency (Cronbach’s alpha = 0.70–0.94). The Turkish PedsQL™ MFS was applied in the study [30].

#### Numerical rating scale (NRS)

The NRS is a scale that uses 11 numbers (ranging from 0 to 10) to measure pain intensity. The patient is asked to select the number that best reflects their pain, with 0 representing “no pain” and 10 representing the “worst (unbearable) pain.” [31]It has been reported that numerical charts from zero to ten can be used in children older than 7–8 years [32].

#### Manual muscle strength test

In the muscle test, the strength of the upper and lower extremity muscles was evaluated by the physiotherapist. The muscle test was conducted using Dr. Robert W. Lovett’s manual muscle testing method [33]. According to this test, Normal (5): The muscle completes full range of motion against gravity with maximum resistance. Good (4): The muscle completes full range of motion against gravity with less than maximum resistance. Fair (3): The muscle completes full range of motion against gravity. Poor (2): The muscle completes full range of motion in a gravity-eliminated position. Trace (1): Muscle contraction is felt without any joint movement. Zero (0): No muscle contraction is felt. A feasibility and reliability study of manual muscle strength testing in children has been conducted [34].

#### Children Depression Rating Scale-Revised (CDRS-R)

The Children’s Depression Inventory (CDI) is a self-assessment scale that can be administered to children aged 6 to 17 years. The inventory consists of 27 items, each offering three different options. The child is asked to choose the statement that best describes how they have felt over the past two weeks. A higher score indicates more severe depression, with a recommended cutoff score of 19 point [35, 36]. The Turkish CDRS-R was applied in the study [37].

#### Timed Up and Go (TUG) test

This test measures mobility, which requires static and dynamic balance. The children were asked to stand up from a chair, walk 3 m with their assistive device, turn around, and walk back to the chair as quickly as possible. The time required to act was timed using a stopwatch [38]. All children were allowed to get support from the arms of the chair while standing and while returning to a sitting position.

### Data analyses

Statistical analyses were performed using SPSS (Version 28) and Python (Version 3.8). The variables were investigated using visual (histograms and probability plots) and analytical methods (Kolmogorov–Smirnov/Shapiro–Wilk’s test) to determine whether they are normally distributed. Mean, standard deviation, and minimum–maximum values were calculated for numerical variables; frequency tables were created for ordinal variables. The logistic regression model was used to evaluate the effect of independent variables on the dependent variable. While creating the regression model, Python programming language was used, and the Stats models library was preferred for statistical calculations and creating the model. Categories obtained from the TSK were used to define the dependent variable. TSK scores were categorized as follows: scores < 37 were considered low kinesiophobia, and scores ≥ 37 were considered high kinesiophobia. This categorization aligns with previous studies, such as Yu et al. (2024), which employed a similar method when evaluating kinesiophobia in children with malignant bone tumors [39]. The following assumptions were considered in logistic regression analysis: (1) binary dependent variable: The dependent variable Tampa category is binary categorical; (2) non-linear relationship: The relationship of independent variables with the dependent variable is not linear; instead, it is modeled with logistic function; (3) independent observations: All observations are independent of each other. The result of one observation in the data set does not affect the other; (4) control of multicollinearity: There should be no multicollinearity (high correlation) problem among independent variables. Highly correlated variables were controlled by correlation analysis and some variables were removed to prevent multicollinearity. Necessary adjustments were made to prevent multicollinearity problems among the variables showing high correlation and the model was created with the following independent variables: surgery status (0: not present, 1: present), BMI, NRS (Pain level), PedsQL™ MFS total (general fatigue status), CDRS-R (depression level). The success rate of the model was evaluated with the McFadden *R*^2^ value. The significance level was accepted as *p* < 0.05. The results were interpreted with the odds ratio (OR). Correlation coefficients were interpreted based on Cohen’s guidelines, where coefficients between 0.10 and 0.29 were considered low, between 0.30 and 0.49 as medium, and ≥ 0.50 as high [40] (Statistical power analysis for the behavioral sciences, Routledge). OR were interpreted such that OR close to 1 indicated a neutral or no effect, OR > 1 suggested an increased likelihood of the outcome, and OR < 1 suggested a decreased likelihood. For instance, an OR = 2 indicated a twofold increase in the likelihood of the outcome [41]. To examine the relationship between kinesiophobia level (Tampa category: 0 = low, 1 = high) and muscle strength, the chi-square test was applied according to categorical muscle test scores. Muscle tests for upper and lower extremity muscles were evaluated categorically. Muscle tests were divided into the following categories: 0 = complete paralysis, 1 = contraction, 2 = weak, 3 = moderate, 4 = good, and 5 = normal. The Pearson chi-square test was used to compare categorical data. The test was applied to determine whether there was a significant relationship between kinesiophobia level and each muscle group. The significance level was accepted as *p* < 0.05.

## Results

A total of 118 children were invited to participate in the study. Children excluded from the study were as follows: those receiving palliative care due to metastatic disease with ongoing treatment processes (*n* = 4), those diagnosed with severe depression (*n* = 3), children with a primary tumor of osteosarcoma but with detected lung metastases (*n* = 2), children who developed osteomyelitis after surgery and whose medical stabilization could not be achieved (*n* = 1), and children or their families who did not volunteer to participate in the study (*n* = 8). The study was completed with 100 children, with a mean age of 11.83 years. Descriptive information of the children and their disease-related information are summarized in Table 1.

**Table 1:** Descriptive information of children with bone tumors (*n* = 100)

	Mean (SD)	Min–max
**Age (years)**	11.83 (1.99)	8–16
**Time after surgery (months)**	11.31 (3.38)	6–17
**BMI**	22.89 (4.31)	15.2–29.9
**NRS (pain)**	5.28 (2)	1–9
**PEDSQOL MFS**		
General fatigue	69.58 (18.03)	41–99
Sleep and rest	69.07 (16.81)	40–99
Cognitive fatigue	71.14 (18.61)	40–98
Total	69.93 (10.25)	45–91.3
**CDRS-R**	32.47 (12.82)	7–53
**TSK**	43.55 (14.06)	20–67
**TUG**	28.09 (15.91)	4.4–77.8
	***n***	**%**
**Gender**		
Male	65	65
Female	35	35
**Tumor type**		
Osteosarcoma	57	57
Ewing sarcoma	43	43
**Surgery status**		
Yes	57	57
No	43	43
**Type of surgery**		
Lower extremity	32	56.1
Upper extremity	22	38.6
Both extremities	3	5.3
**Types of treatment received**		
Chemotherapy	34	34
Radiotherapy	33	33
Combined	33	33
**Falling story**		
Yes	15	15
No	85	85
**Assistive device/orthosis usage status**		
Yes	38	38
No	62	62
**Tampa category**		
0 ( < 37)	36	36
1 (˃37)	64	64

*BMI*, body mass index; *NRS*, numerical rating scale; *PEDSQOL MFS*, Pediatrik Quality of Life Inventory Multidimensional Fatigue Scale; *CDRS*-*R*, Children Depression Rating Scale-Revised; *TSK*, Tampa Scale of Kinesiophobia; *TUG*, Time and Up Go Test; Tampa category, 0; low kinesiophobia 1, high kinesiophobia

The mean PedsQL MFS total score was 69.93 (*SD* = 10.25). The mean score on the CDRS-R was 32.47 (*SD* = 12.82). The mean TSK score was 43.55 (*SD* = 14.06), and the mean TUG time was 28.09 s (*SD* = 15.91), with a range of 4.4–77.8 s. Additionally, 15% of participants reported a history of falls, and 38% used assistive devices or orthoses. Kinesiophobia levels were categorized as low (TSK < 37) in 36% of participants and high (TSK > 37) in 64%.

**Fig. 1:**
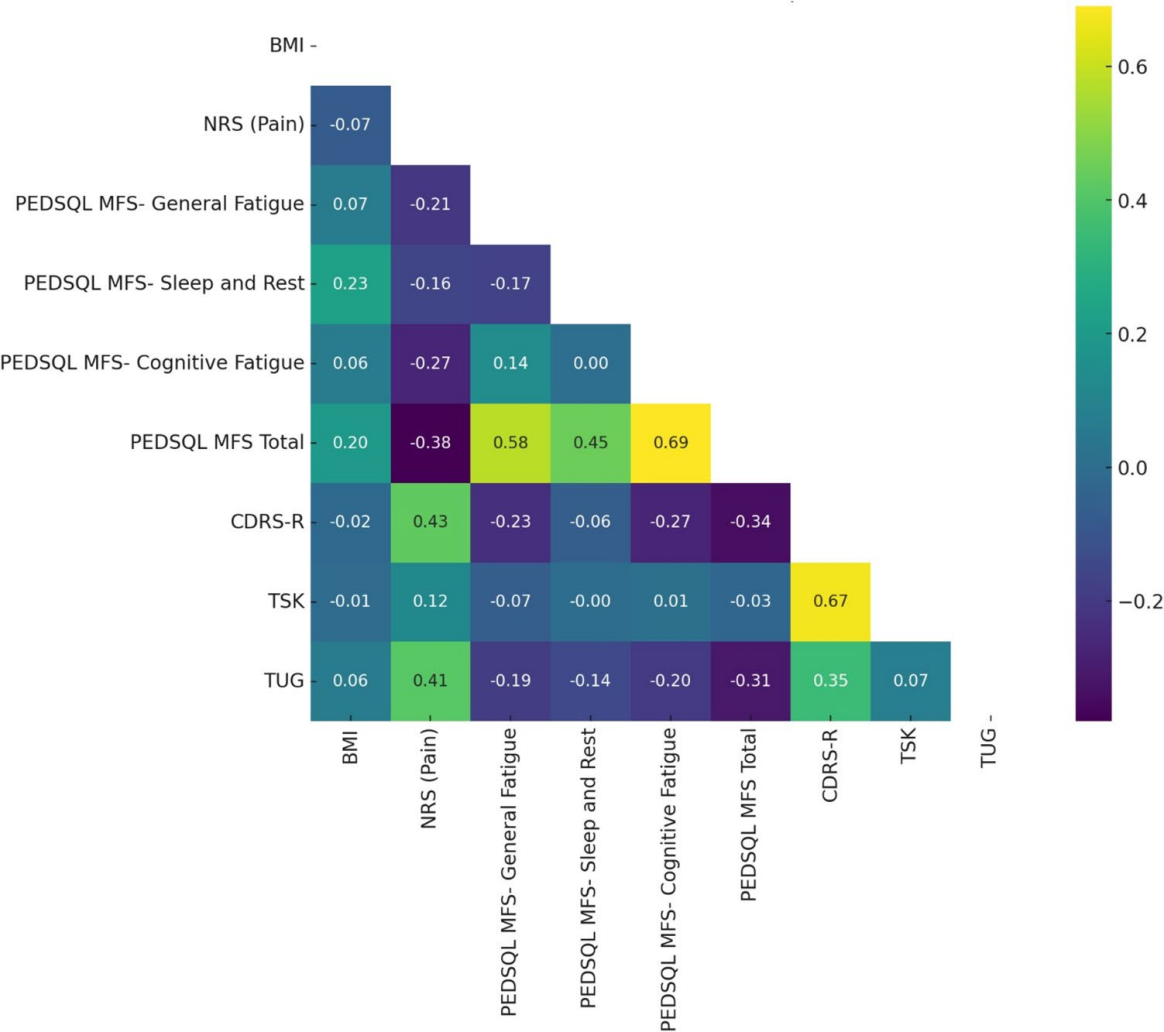
Pearson correlation matrix. BMI: body mass index; NRS: numerical rating scale; PEDSQOL MFS: Pediatrik Quality of Life Inventory Multidimensional Fatigue Scale; CDRS-R: Children Depression Rating Scale-Revised; TSK: Tampa Scale of Kinesiophobia; TUG: Timed and Up Go test

Before creating the logistic regression model, the relationship between the variables was examined. The correlation analysis results are shown in Fig. 1.

General fatigue, sleep and rest, and cognitive fatigue represent subscales of the same test (PedsQL™ MFS). When the correlation analysis is examined, a high correlation is seen between these subscales ((between general fatigue and total score *r* = 0.58 (*p* < 0.001); between cognitive fatigue and total score *r* = 0.69 (*p* < 0.001). These strong relationships may cause multicollinearity and disrupt the stability of the model. Therefore, only the total score among these subscales was kept in the model and the other subscales were removed. Using the total score will be sufficient to represent a general effect related to fatigue and will prevent multicollinearity.

There are also significant correlations between TUG test results and some other variables. There is a relationship between TUG and NRS (pain) of *r* = 0.41 (*p* < 0.001); and between TUG and general fatigue of *r* =  − 0.19 (*p* = 0.057). These relationships may lead to a multicollinearity problem if TUG is added to the model. Therefore, it became necessary to remove TUG from the regression model. OR values were used to interpret the effects of surgery status, pain levels, and fatigue on kinesiophobia. An OR > 1 indicates an increased likelihood of kinesiophobia, while an OR < 1 suggests a decreased likelihood. The OR for surgery status was found to be 1420.82, indicating that children who underwent surgery had a significantly higher likelihood of developing kinesiophobia compared to those who did not. Similarly, the OR for NRS pain levels was 5.35, suggesting that a one-unit increase in pain level increased the likelihood of kinesiophobia by more than five times. Conversely, the OR for fatigue, as measured by the PedsQL™ Multidimensional Fatigue Scale total score, was 0.69, indicating that higher fatigue levels were associated with a reduced likelihood of kinesiophobia. The model became more stable and gave a significant result when this variable was removed. The results of the logistic regression model are shown in Table 2.

**Table 2:** Results of logistic regression analysis (dependent variable kinesiophobia category 0: low, 1: high)

Predictor	*β*	SE	*p*	Odds ratio
Constant	6.02	8.03	0.45	412.55
Surgery	7.25	3.03	0.02*	1420.82
BMI	0.05	0.17	0.73	1.06
NRS (pain)	1.67	0.76	0.03*	5.35
PEDSQOL MFS Total	− 0.36	0.17	0.04*	0.69
CDRS-R	0.29	0.11	0.008*	1.34

*β*, coefficient; *SE*, standard error; *BMI*, body mass index; *NRS*, numerical rating scale; *PEDSQOL MFS*, Pediatric Quality of Life Inventory Multidimensional Fatigue Scale; *CDRS*-*R*, Children Depression Rating Scale-Revised; *p < 0.05

**Fig. 2:**
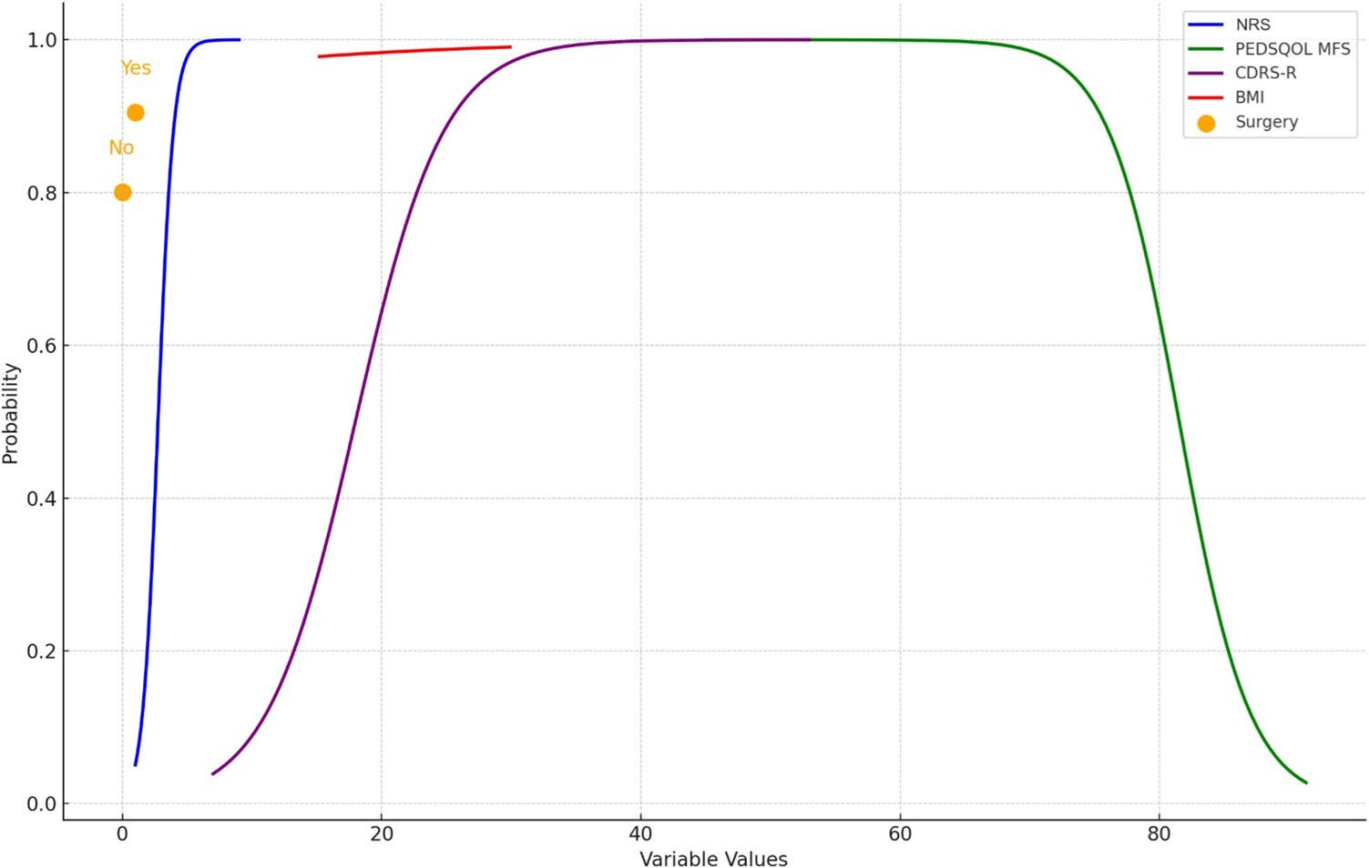
Predicted probabilities by variable levels for “Category of Kinesiophobia = 1”

The McFadden *R*^2^ value for the logistic regression model was calculated as 0.870. This value shows the explanatory power of the model for the dependent variable Tampa category (kinesiophobia level) and it is seen that the model explains 87% of the kinesiophobia level. A McFadden *R*^2^ of over 0.4 is considered a good model. In this context, the obtained value shows that the model has a very high explanatory power. The variables that have a significant effect on the kinesiophobia level are summarized in Fig. 2.

According to the results of the chi-square test, significant relationships were found between some muscle strength values of children with bone tumors and the level of kinesiophobia, but for most muscle groups, *p* values were above 0.05 and statistical significance was not found. The results are summarized in Table 3.

**Table 3:** The relationship between kinesiophobia level (0: low, 1: high) and muscle strength of children with bone tumors

	***x***^**2**^	***p***
**Shoulder**		
Flexor right	0.01	0.91
Flexor left	0.01	0.91
Extensor right	0.14	0.71
Extensor left	0.14	0.71
Abductor right	1.92	0.38
Abductor left	0.09	0.75
Adductor right	0.09	0.75
Adductor left	0.09	0.75
**Elbow**		
Flexor right	0.46	0.49
Flexor left	0.46	0.49
Extensor right	3.24	0.07
Extensor left	3.24	0.07
**Forearm**		
Pronator right	4.27	0.03*
Pronator left	4.27	0.03*
Supinator right	2.43	0.11
Supinator left	2.43	0.11
**Hip**		
Extensors	0.03	0.85
Flexors	2.03	0.15
Abductors	6.68	0.03*
Adductors	6.68	0.03*
**Knee**		
Flexor right	18.73	0.0003***
Flexor left	15.55	0.001**
Extensor right	35.28	< 0.001***
Extensor left	33.88	< 0.001***
**Ankle**		
Dorsiflexor right	1.99	0.36
Dorsiflexor left	1.14	0.28
Plantarflexor right	0.14	0.71
Plantarflexor left	0.14	0.71

^*^p < 0.05; **p < 0.01; ***p < 0.001

## Discussion

This study cross-sectionally examined the presence of kinesiophobia and associated factors in the pediatric population with malignant bone tumors. The study findings highlight the complex interaction between such as kinesiphobia, psychological factors, and physical health outcomes in children with bone tumors.

The presence of kinesiophobia related to surgical processes has been reported in the literature for pediatric populations [24, 42]. Additionally, the presence and risk of kinesiophobia in adult cancer patients are available in the literature [21, 43]. The presence of kinesiphobia in pediatric cancer patients is a relatively new topic. Although kinesiophobia has primarily been studied in adult populations, it may be a multifactorial issue in children, potentially affecting movement and, consequently, functionality. Diekfuss et al. [44] reported the presence of kinesiophobia in children with patellofemoral pain, while Alkan et al. [45] highlighted kinesiophobia in children with Duchenne muscular dystrophy. Similarly, Ye et al. [46] documented the presence of kinesiophobia and its associated factors in adolescent scoliosis patients undergoing corrective surgery. There is limited research in the literature specifically addressing kinesiophobia in children with cancer, particularly those with malignant tumors affecting the musculoskeletal system [39]. Yu et al. [39] identified high levels of kinesiophobia in 89% of children with bone tumors during the postoperative period. However, this study did not examine kinesiphobia directly but as an outcome variable. This study confirmed the presence of high kinesiophobia in 64% of children with bone tumors.

The McFadden *R*^2^ value was calculated as 0.8707, indicating that the model explains 87% of the variance in kinesiophobia levels. McFadden *R*^2^ values above 0.4 are generally considered strong models in behavioral and health-related research [47]. The key variables identified in this study—surgical status, pain levels (NRS), and fatigue—align with previous research highlighting the role of physical health, pain, and psychological factors in determining fear of movement [48].

The relationship between surgery and kinesiophobia is particularly noteworthy in pediatric oncology patients. The findings of this study revealed that children who underwent surgery exhibited higher levels of kinesiophobia compared to those who did not. Several factors may contribute to the development of kinesiophobia following surgery. One of the most significant contributors is pain. Pain reinforces the perception that movement could potentially cause greater discomfort, leading to movement avoidance and, ultimately, kinesiophobia [39]. Additionally, the relationship between depression levels and kinesiophobia, the capacity to cope with pain after surgery, psychological resilience, and the type of surgery undergone may influence the severity of kinesiophobia [49]. This finding aligns with previous studies addressing the relationship between surgical intervention and kinesiophobia. Recent studies by Rosenbloom et al. [22] and Ceniza-Bordallo et al. [15] analyzed kinesiophobia in surgical cohorts but did not find a significant association between kinesiophobia and chronic postsurgical pain. In contrast, our study identified a significant relationship, suggesting that kinesiophobia may develop independently of chronic pain in specific contexts, particularly in pediatric oncology patients with malignant bone tumors. This discrepancy may be attributed to the unique characteristics of our study population, where the interplay between surgical trauma, acute pain, and the psychological burden of cancer may amplify fear of movement. While Rosenbloom and Ceniza-Bordallo examined long-term outcomes, our study adopted a cross-sectional approach as it included children who had not undergone surgery. Additionally, the minimum postoperative duration for the children who underwent surgery in our study was 6 months. Therefore, it can be stated that this study similarly focuses on long-term effects. Our findings also align with those of Yu et al. [39], who reported a relationship between pain and kinesiophobia in children with pediatric bone tumors. Although surgery emerged as the most significant factor, the presence of kinesiophobia in children who did not undergo surgery suggests that musculoskeletal system-affecting tumors and the associated treatment processes might contribute to the development of kinesiophobia, potentially as a new “symptom.”

Children with higher depression scores exhibit more severe kinesiophobia. Karen et al. highlighted the interaction between psychological factors and pain perception in cancer patients [50] and this may potentially explain the role of kinesiophobia as a barrier to rehabilitation. The fear-avoidance model [51] is a cognitive-behavioral model that highlights the roles of catastrophizing and kinesiophobia in promoting disability and distress in individuals with chronic pain. The affected emotional state may trigger this cycle in children, leading to heightened kinesiophobia.

Interestingly, our study found a negative relationship between fatigue and kinesiophobia (OR = 0.69), suggesting that higher fatigue levels may reduce the likelihood of kinesiophobia. The relationship between fatigue and kinesiophobia is complex. Some studies describe strong associations between fatigue and kinesiophobia [16], while others report that this association is not predictive [52]. This counterintuitive finding could stem from decreased physical activity and movement in fatigued children, potentially limiting the exposure to situations that provoke movement fear. Velthuis et al. [53] did not find an association between kinesiophobia and fatigue in cancer survivors, attributing this to the multifactorial origins of cancer-related fatigue. They emphasized that fatigue in cancer survivors likely arises from a combination of physical, psychological, and treatment-related factors, making it distinct from the mechanisms that drive kinesiophobia. This finding highlights the complexity of cancer-related fatigue and suggests that it may not always correlate directly with fear of movement, as other underlying factors may play a more dominant role in influencing fatigue. However, this relationship requires further investigation, as the interplay between fatigue, activity levels, and psychological factors in pediatric populations is not well understood.

The relationship between muscle strength and kinesiophobia, however, is less clear. While significant relationships were found between certain muscle groups and kinesiophobia, most muscle groups did not show statistical significance. This finding might be unexpected, as muscle weakness is presumed to contribute to fear of movement due to perceived physical vulnerability [54]. However, the inconsistent relationships between muscle strength and kinesiophobia may be explained by variations in disease progression, treatment regimens, and individual patient characteristics. Additionally, muscle strength alone may not fully capture the complexity of physical functionality in pediatric oncology patients; factors such as endurance, coordination, and flexibility may also play critical roles in functional recovery [55].

In tumors affecting the musculoskeletal system, reduced physical activity levels may be observed; however, this study raises the possibility of a severe clinical condition—kinesiophobia—in children with malignant bone tumors, characterized by fear and avoidance of movement. Using a prospective cross-sectional design, this study aimed to describe the current clinical scenario. Nevertheless, there are some limitations. To clarify the temporal course of interactions among kinesiophobia, pain, and psychological factors, as well as possible causal mechanisms, prospective and long-term follow-up studies are needed. Furthermore, identifying preoperative factors and kinesiophobia levels in children undergoing surgery may lead to a clearer understanding of this issue. Future studies could also examine the multidimensional nature of fatigue and its relationship with kinesiophobia.

## Conclusion

This study provides new insights into the multifactorial nature of kinesiophobia in pediatric oncology children with malignant bone tumors. The findings underscore the significant impact of surgical interventions, pain, fatigue, and psychological factors on the development of kinesiophobia in this vulnerable population. Including psychological, physical, and functional assessments in routine care can help identify children at risk of kinesiophobia early in their treatment journey. Multidisciplinary approaches addressing pain management, psychological support, and physical rehabilitation are essential for reducing kinesiophobia and improving recovery outcomes. In conclusion, this study underscores the importance of addressing kinesiophobia as a multifaceted clinical challenge in children with malignant bone tumors. By adopting a biopsychosocial approach, healthcare providers can better support these children in overcoming movement fear, thereby enhancing their functional outcomes and overall well-being.

## Data Availability

No datasets were generated or analysed during the current study.

## References

[1] Choi JH, Ro JY (2021) The 2020 WHO classification of tumors of bone: an updated review. Adv Anat Pathol 28(3):119–138 33480599 10.1097/PAP.0000000000000293

[2] Whelan JS, Davis LE (2018) Osteosarcoma, chondrosarcoma, and chordoma. J Clin Oncol 36(2):188–193 29220289 10.1200/JCO.2017.75.1743

[3] Kutluk T (2024) Çocukluk Çağı Kanserlerinin Epidemiyolojisi ve Yaşam Hızları. Turkiye Klinikleri Pediatric Hematology and Oncology-Special Topics 5(2):5–8

[4] Ferguson JL, Turner SP (2018) Bone cancer: diagnosis and treatment principles. Am Fam Physician 98(4):205–213 30215968

[5] Kebudi R, Emiroglu HH, Gorgun O, Akici F, Tugcu D, Inci A (2012) Pediatric malignant liver tumors: results of a single center. Int J Hematol Oncol 34(1):001–008

[6] Erdmann F, Frederiksen LE, Bonaventure A et al (2021) Childhood cancer: survival, treatment modalities, late effects and improvements over time. Cancer Epidemiol 71:101733 32461035 10.1016/j.canep.2020.101733

[7] Kudubeş AA, Bektaş M (2017) Pediatrik Onkoloji Hastalarında Yorgunluğun Yaşam Kalitesine Etkisi. J Pediatric Res 4(3)

[8] Yu Q, Fang F, Chen L, Wang Q, Dai W. The relationship of pain catastrophizing in principal caregivers of postoperative children with malignant bone tumors and children’s kinesiophobia and nonciception: a cross-sectional survey. Available at SSRN 4751741. 10.1016/j.ijotn.2024.101137 39307042

[9] Gerbershagen HJ, Aduckathil S, van Wijck AJ, Peelen LM, Kalkman CJ, Meissner W (2013) Pain intensity on the first day after surgery: a prospective cohort study comparing 179 surgical procedures. Anesthesiology 118(4):934–944 23392233 10.1097/ALN.0b013e31828866b3

[10] Sevinir B (2004) Çocuklarda kanser ve ağrı. Güncel Pediatri 2(2):103–108

[11] Finnegan L (2007) Correlates of physical activity in young adult survivors of childhood cancers. Number 5/September 2007 34(5):E60-E69. 10.1188/07.ONF.E60-E69 17878118

[12] Roelofs J, Van Breukelen G, Sluiter J et al (2011) Norming of the Tampa Scale for Kinesiophobia across pain diagnoses and various countries. Pain 152(5):1090–1095 21444153 10.1016/j.pain.2011.01.028

[13] Malchrowicz-Mośko E, Waśkiewicz Z, Castañeda-Babarro A, León-Guereño P (2024) Kinesiophobia–psychological aspects of physical activity in breast cancer patients. Frontiers Media SA 1380019. 10.3389/fpsyg.2024.1380019 PMC10910011 38440239

[14] Sh K (1990) Kinesiophobia: a new view of chronic pain behavior. Pain Manage 3:35–43

[15] Ceniza-Bordallo G, Fraile AG, Martín-Casas P et al (2025) Prevalence, pain trajectories, and presurgical predictors for chronic postsurgical pain in a pediatric sample in Spain with a 24-month follow-up. Pain 166(1):112–122 39047258 10.1097/j.pain.0000000000003330 PMC11856900

[16] Sunar İ, Sunar V (2021) Kinesiophobia in breast cancer survivors and its relationship with quality of life, comorbidity, and other clinical parameters. Acta Oncologica Turcica (54)

[17] Cruz-Díaz D, Romeu M, Velasco-González C, Martínez-Amat A, Hita-Contreras F (2018) The effectiveness of 12 weeks of Pilates intervention on disability, pain and kinesiophobia in patients with chronic low back pain: a randomized controlled trial. Clin Rehabil 32(9):1249–1257 29651872 10.1177/0269215518768393

[18] Luque-Suarez A, Martinez-Calderon J, Falla D (2019) Role of kinesiophobia on pain, disability and quality of life in people suffering from chronic musculoskeletal pain: a systematic review. Br J Sports Med 53(9):554–559 29666064 10.1136/bjsports-2017-098673

[19] Sunar İ, Sunar V. Kinesiophobia in breast cancer survivors and its relationship with quality of life, comorbidity and other clinical parameters Meme Kanseri Hastalarında Kinezyofobinin Hayat Kalitesi, Komorbidite ve Diğer Klinik Özellikler ile İlişkisi.

[20] Van der Gucht E, Dams L, Meeus M et al (2020) Kinesiophobia contributes to pain-related disability in breast cancer survivors: a cross-sectional study. Support Care Cancer 28:4501–4508 31953624 10.1007/s00520-020-05304-4

[21] Can AG, Can SS, Ekşioğlu E, Çakcı FA (2019) Is kinesiophobia associated with lymphedema, upper extremity function, and psychological morbidity in breast cancer survivors? Turkish J Phys Med Rehab 65(2):139 10.5606/tftrd.2019.2585 PMC6706825 31453554

[22] Rosenbloom BN, Pagé MG, Isaac L et al (2019) Pediatric chronic postsurgical pain and functional disability: a prospective study of risk factors up to one year after major surgery. J Pain Res 12:3079–3098 31814752 10.2147/JPR.S210594 PMC6858804

[23] Velthuis MJ, Peeters PH, Gijsen BC et al (2012) Role of fear of movement in cancer survivors participating in a rehabilitation program: a longitudinal cohort study. Arch Phys Med Rehabil 93(2):332–338 22289246 10.1016/j.apmr.2011.08.014

[24] Woolnough LU, Lentini L, Sharififar S, Chen C, Vincent HK (2022) The relationships of kinesiophobia and physical function and physical activity level in juvenile idiopathic arthritis. Pediatr Rheumatol 20(1):73 10.1186/s12969-022-00734-2 PMC9438303 36050703

[25] Swinkels-Meewisse E, Swinkels R, Verbeek A, Vlaeyen J, Oostendorp R (2003) Psychometric properties of the Tampa Scale for kinesiophobia and the fear-avoidance beliefs questionnaire in acute low back pain. Man Ther 8(1):29–36 12586559 10.1054/math.2002.0484

[26] Vlaeyen JW, Kole-Snijders AM, Boeren RG, Van Eek H (1995) Fear of movement/(re) injury in chronic low back pain and its relation to behavioral performance. Pain 62(3):363–372 8657437 10.1016/0304-3959(94)00279-N

[27] Rosenbloom BN, Pagé MG, Isaac L et al (2020) Fear of movement in children and adolescents undergoing major surgery: a psychometric evaluation of the Tampa Scale for Kinesiophobia. Eur J Pain 24(10):1999–2014 32761986 10.1002/ejp.1643

[28] Yilmaz O, Yakut Y, Uygur F, Ulug N (2011) Turkish version of the Tampa Scale for Kinesiophobia and its test-retest reliability. Turkish J Physiother Rehabilitation-Fizyoterapi Rehabilitasyon 22(1)

[29] Varni JW, Burwinkle TM, Katz ER, Meeske K, Dickinson P (2002) The PedsQL™ in pediatric cancer: reliability and validity of the pediatric quality of life inventory™ generic core scales, multidimensional fatigue scale, and cancer module. Cancer 94(7):2090–2106 11932914 10.1002/cncr.10428

[30] Alemdaroglu-Gürbüz I, Bulut N, Bozgeyik S et al (2019) Reliability and validity of the turkish translation of pedsql™ multidimensional Fatigue scale in Duchenne Muscular Dystrophy. Neurosci J 24(4):302–310 10.17712/nsj.2019.4.20190035 PMC8015545 31872810

[31] Farrar JT, Young JP Jr, LaMoreaux L, Werth JL, Poole RM (2001) Clinical importance of changes in chronic pain intensity measured on an 11-point numerical pain rating scale. Pain 94(2):149–158 11690728 10.1016/S0304-3959(01)00349-9

[32] Gehdoo R (2004) Post operative pain management in paediatric patients. Indian J Anaesth 48(5):406–414

[33] Lovett RW, Martin EG (1916) Certain aspects of infantile paralysis: with a description of a method of muscle testing. J Am Med Assoc 66(10):729–733

[34] Siu K, Al-Harbi S, Clark H et al (2015) Feasibility and reliability of muscle strength testing in critically ill children. J Pediatric Intensive Care 4(04):218–224 10.1055/s-0035-1563544 PMC6513132 31110873

[35] Keller F, Grieb J, Ernst M, Spröber N, Fegert JM, Kölch M (2011) Children’s depression rating scale–revised (CDRS-R). Zeitschrift für Kinder-und Jugendpsychiatrie und Psychotherapie 10.1024/1422-4917/a000090 21563109

[36] Güney SA, Baykara HB, Emiroğlu NI (2018) Çocuklar için Depresyon Değerlendirme Ölçeği Revize Formunun Türk popülasyonundaki ergenlerde psikometrik özellikleri. Anatolian Journal of Psychiatry/Anadolu Psikiyatri Dergisi 19(1)

[37] Guney SA, Baykara HB, Emiroglu NI (2018) Psychometric properties of the Turkish adaptation of the Children’s Depression Rating Scale: revised in Turkish adolescents/Cocuklar icin Depresyon Degerlendirme Olcegi Revize Formunun Turk populasyonundaki ergenlerde psikometrik ozellikleri. Anadolu Psikiyatri Dergisi 19(S1):41–49

[38] Podsiadlo D, Richardson S (1991) The timed “Up & Go”: a test of basic functional mobility for frail elderly persons. J Am Geriatr Soc 39(2):142–148 1991946 10.1111/j.1532-5415.1991.tb01616.x

[39] Yu Q, Fang F, Chen L, Wang Q, Dai W (2024) The relationship of pain catastrophizing in principal caregivers of postoperative children with malignant bone tumors and children’s kinesiophobia and pain perception: a cross-sectional survey. Int J Orthopaedic Trauma Nurs 55:101137 10.1016/j.ijotn.2024.101137 39307042

[40] Cohen J (2013) Statistical power analysis for the behavioral sciences. routledge

[41] Borenstein M, Hedges LV, Higgins JP, Rothstein HR (2021) Introduction to meta-analysis. John Wiley & Sons.

[42] Diekfuss JA, Grooms DR, Coghill RC et al (2020) Kinesiophobia is related to brain activity for knee motor control in pediatric patients with patellofemoral pain. Orthopaedic J Sports Med 8(4_suppl3):2325967120S00187

[43] Malchrowicz-Mośko E, Nowaczyk P, Wasiewicz J et al (2023) The level of kinesiophobia in breast cancer women undergoing surgical treatment. Front Oncol 13:1010315 36816937 10.3389/fonc.2023.1010315 PMC9932589

[44] Diekfuss JA, Foss KDB, Slutsky-Ganesh AB et al (2021) Does fear of movement alter brain activity? Investigating the neural markers of kinesiophobia in pediatric patients with patellofemoral pain. Orthopaedic J Sports Med 9(7_suppl3):2325967121S00122

[45] Alkan H, Bulut N, Özel CB et al (2015) Kinesiophobia in Duchenne muscular dystrophy: perspective of parents and physiotherapists. Neuromuscul Disord 25:S304–S305

[46] Ye D, Bote S, Ouellet J, Ferland C (2018) Preliminary analysis of a novel objective assessment of kinesiophobia in adolescent scoliosis patients scheduled for corrective surgery. J Pain 19(3):S40

[47] McFadden D (1974) The measurement of urban travel demand. J Public Econ 3(4):303–328

[48] Martinez-Calderon J, Meeus M, Struyf F, Morales-Asencio JM, Gijon-Nogueron G, Luque-Suarez A (2018) The role of psychological factors in the perpetuation of pain intensity and disability in people with chronic shoulder pain: a systematic review. BMJ Open 8(4):e020703 29654040 10.1136/bmjopen-2017-020703 PMC5905738

[49] McGarrigle L, Wesson C, DeAmicis L, Connoly S, Ferreira N (2020) Psychological mediators in the relationship between paediatric chronic pain and adjustment: an investigation of acceptance, catastrophising and kinesiophobia. J Contextual Behav Sci 18:294–305

[50] Syrjala KL, Jensen MP, Mendoza ME, Yi JC, Fisher HM, Keefe FJ (2014) Psychological and behavioral approaches to cancer pain management. J Clin Oncol 32(16):1703–1711 24799497 10.1200/JCO.2013.54.4825 PMC4031190

[51] Vlaeyen JW, Linton SJ (2000) Fear-avoidance and its consequences in chronic musculoskeletal pain: a state of the art. Pain 85(3):317–332 10781906 10.1016/S0304-3959(99)00242-0

[52] Nijs J, Meeus M, Heins M, Knoop H, Moorkens G, Bleijenberg G (2012) Kinesiophobia, catastrophizing and anticipated symptoms before stair climbing in chronic fatigue syndrome: an experimental study. Disabil Rehabil 34(15):1299–1305 22324510 10.3109/09638288.2011.641661

[53] Velthuis MJ, Van den Bussche E, May AM, Gijsen BC, Nijs S, Vlaeyen JW (2012) Fear of movement in cancer survivors: validation of the Modified Tampa Scale of Kinesiophobia—Fatigue. Psychooncology 21(7):762–770 21538679 10.1002/pon.1971

[54] Aydemir B, Huang CH, Foucher KC (2022) Strength and physical activity in osteoarthritis: the mediating role of kinesiophobia. J Orthopaedic Res® 40(5):1135–1142 10.1002/jor.25151 PMC8799782 34324222

[55] Söntgerath R, Däggelmann J, Kesting SV et al (2022) Physical and functional performance assessment in pediatric oncology: a systematic review. Pediatr Res 91(4):743–756 33859367 10.1038/s41390-021-01523-5 PMC9064803

